# An Unusual Presentation of a Rare Disease: Eosinophilic Fasciitis

**DOI:** 10.7759/cureus.31140

**Published:** 2022-11-05

**Authors:** Shubham Dakhode, Rudra M Prabhu, Sanjay K Barik, Furquan Ulhaque, Abhishek Kumar Rai

**Affiliations:** 1 Orthopedics, Topiwala National Medical College & B. Y. L. Nair Charitable Hospital, Mumbai, IND; 2 Orthopedics, King Edward Memorial Hospital and Seth Gordhandas Sunderdas Medical College, Mumbai, IND; 3 Orthopedics, Mahatma Gandhi Institute of Medical Sciences, Sevagram, Wardha, IND

**Keywords:** corticosteroids, biopsy, recurrence, eosinophilic fasciitis, forearm

## Abstract

Eosinophilic fasciitis is an uncommon disorder presenting with diffuse fasciitis and peripheral eosinophilia. Due to the rarity of this disorder and limited literature, its diagnosis and treatment are often delayed. We present the case of a young male wherein the diagnosis of eosinophilic fasciitis was initially delayed due to an atypical presentation. However, after the diagnosis was confirmed, the patient was successfully managed with oral corticosteroids. A well-written informed consent was obtained from the patient prior to the preparation of this manuscript.

An 18-year-old right-hand dominant male presented with a sudden onset, progressive, non-traumatic, left forearm swelling associated with a weak hand grip. The swelling was tender on examination with a local rise in temperature. Radiographs taken at the time of presentation revealed no osseous pathology. As the initial blood investigations were suggestive of a localized inflammatory pathology involving the forearm, the patient was managed with non-steroidal anti-inflammatory drugs and analgesics. He returned 6 months later with a recurrence of the symptoms. A magnetic resonance imaging scan of the left forearm was performed to further investigate the pathology and it was suggestive of a diffuse plaque-like thickening involving the myofascial layer of the muscles. Blood investigations showed peripheral eosinophilia, raised immunoglobulin G count, and raised inflammatory markers. A full-thickness forearm biopsy showed the presence of lymphocytic infiltration. A diagnosis of eosinophilic fasciitis was suspected and the patient was managed with oral corticosteroids, given as a tapering dose. Following this, the patient had symptomatic improvement with the resolution of the deranged blood parameters. He was asymptomatic at the latest follow-up of 4 years.

## Introduction

The age of onset of eosinophilic fasciitis (EF) ranges from 40 to 50 years with women and Caucasians being more affected [1, 2]. Disorders of the immune system cause this condition and it has shown an association with rheumatoid arthritis and hypergammaglobulinemia [3].

In the absence of well-established criteria, the diagnosis of the disease is often missed. As patients can present with symptoms such as swelling, pain, and erythema, they can mimic symptoms resulting from blunt trauma to the limb and can be referred to the orthopedic surgeon during the initial stages and if the correct intervention is initiated at this point, the morbidity associated with the disease is significantly reduced.

## Case presentation

**Figure 1:**
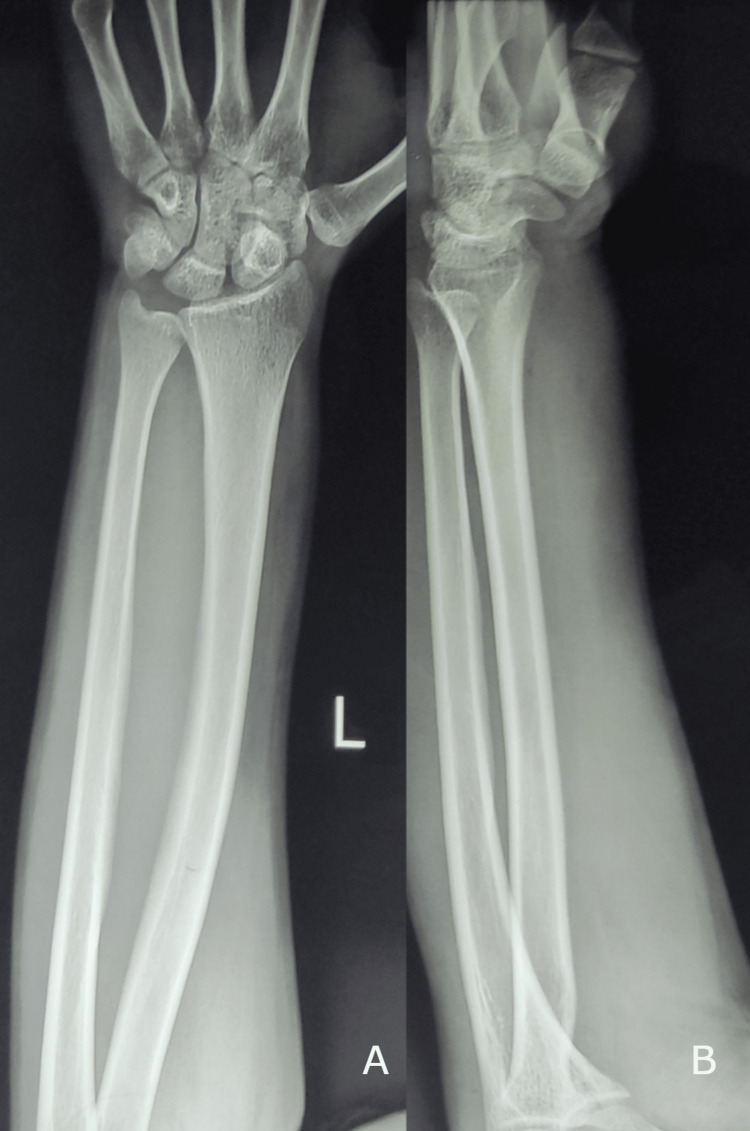
Radiographs of the forearm and wrist revealed no significant abnormality

An 18-year-old right-hand dominant male student presented to our emergency department with a swelling of the left forearm for 1 week. The swelling had an acute onset and was progressive in nature. The patient complained of difficulty in gripping objects using his left hand. There was no history of trauma, the family history was not significant, and the patient did not have any other medical condition of significance. There was no previous history of a similar episode in the same limb or other extremities. On inspection, the patient had a diffuse erythematous swelling involving the forearm along with a prominence of the veins. There were no visible pulsations or any associated wasting involving the forearm. On palpation, there was local tenderness along with a local rise in temperature. The skin over the forearm was indurated and free from the underlying structures and the swelling was not reducible on palpation. There was no evidence of any distal neurovascular deficit. Initial radiographs of the forearm and hand were normal (Figure 1A-1B). 

The lab investigations revealed a raised white blood cell count of 12,500 per mm^3 ^(normal range, 4000-11000 per mm^3^), the erythrocyte sedimentation rate was 58 mm/hour (normal range, 0-22 mm/hour), and the C-reactive protein level was 72 mg/L (normal <10 mg/L). Other investigations were within normal limits. As no formal diagnosis other than that of a localized inflammatory pathology or a traumatic event to the forearm could be formulated at this point, the patient was admitted for observation and was given an injection of paracetamol intravenously at a dose of 15 mg/kg body weight every 6 hours for the initial 24 hours. This was followed by a combination of oral aceclofenac 100 mg and paracetamol 325 mg twice a day.

**Figure 2:**
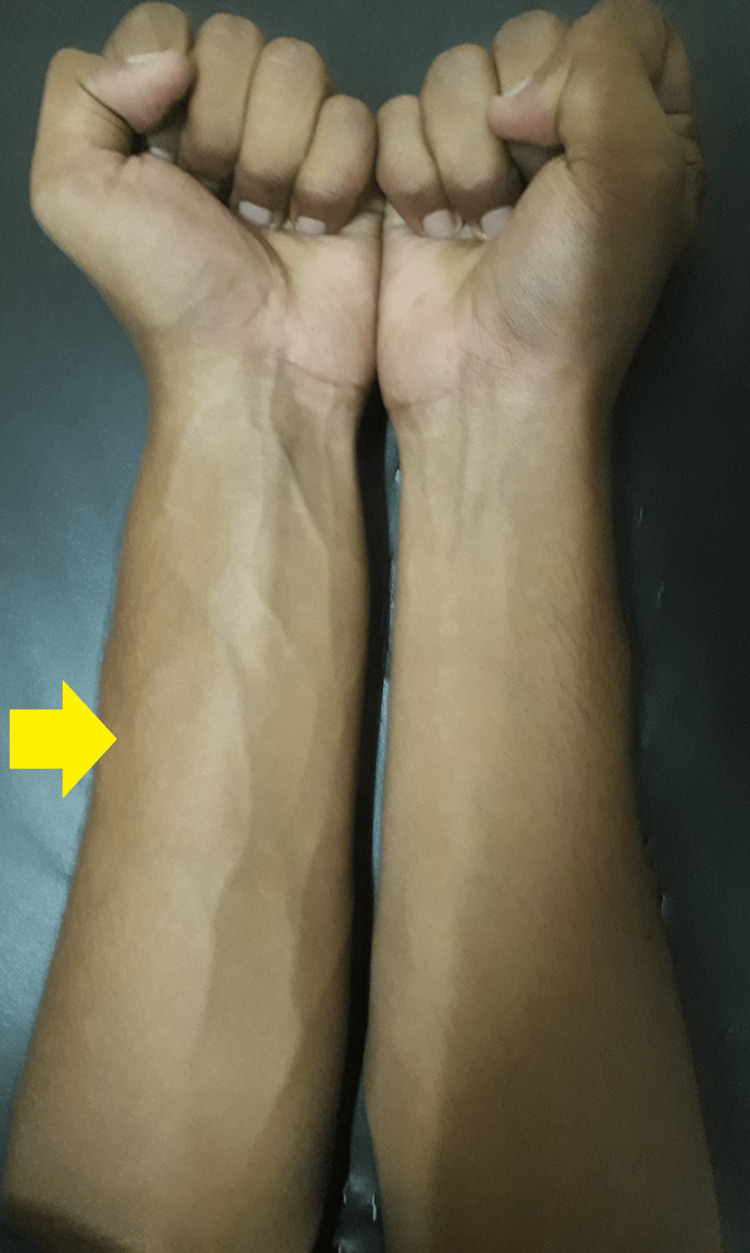
Clinical image at the time of recurrence of symptoms shows a swollen left forearm with prominent veins (yellow arrow) in comparison to the right.

The patient showed a response to treatment with a decrease in swelling, redness, and local temperature after around 10 days of treatment. His white blood count cell count decreased to 10,000 per mm^3^. The erythrocyte sedimentation rate fell to 32 mm/hour and the C-reactive protein level decreased to 38 mg/L. As the patient had an improvement in his clinical and lab parameters, he was discharged and asked to follow up after 2 weeks with fresh blood investigations or earlier if he had any recurrence of his symptoms. However, the patient did not follow up regularly but returned to our center around 6-7 months later with a recurrence of the symptoms and severe pain involving the left forearm. The symptoms were acute in onset. He gave a history of having intermittent flare-ups with acute onset of symptoms involving the left forearm during the past 6 months and a temporary resolution with non-steroidal anti-inflammatory drugs. The patient recalled that he had approximately four to six episodes in the past 6 months. However, this time he had severe pain along with weakness in his left-hand grip that was not responding to non-steroidal anti-inflammatory drugs, and thus, he presented to our center (Figure 2).

**Figure 3:**
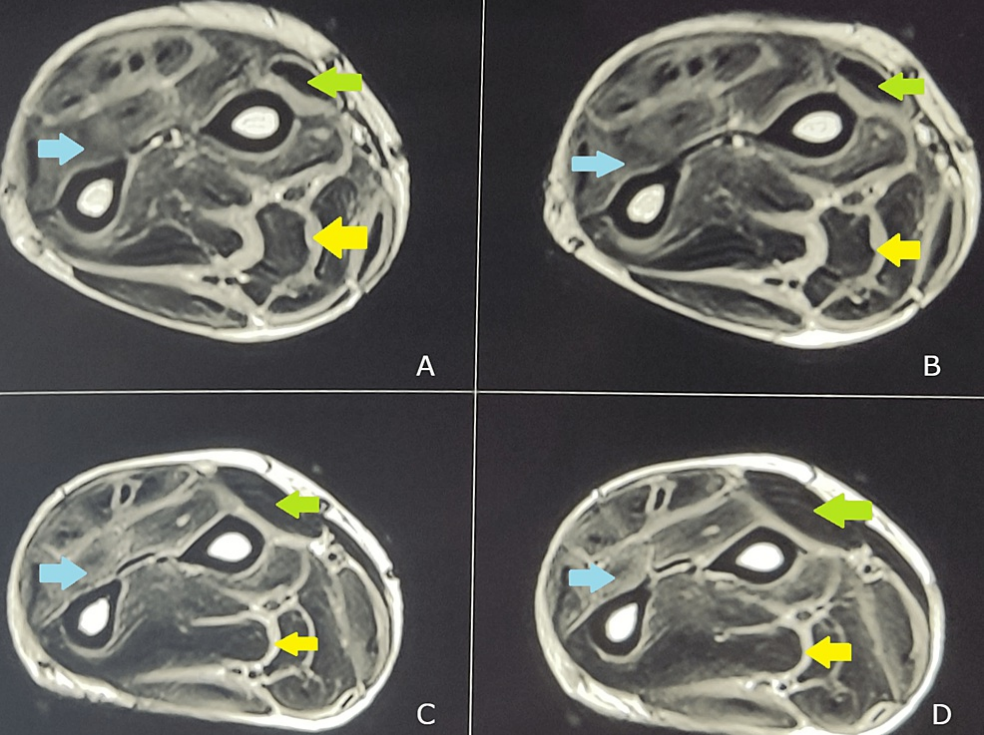
Axial magnetic resonance imaging scan sections showing the presence of an inflammatory pathology The axial sections show the obliteration of intermuscular fascial planes with thickening (yellow arrow). Due to the presence of an inflammatory pathology, the muscles appear whiter than normal (blue arrow). The relatively spared muscles that appear darker have been marked for comparison (green arrow).

To further investigate the pathology, a magnetic resonance imaging (MRI) scan of the left forearm was performed. It showed the presence of a diffuse plaque-like thickening involving the myofascial layer of the muscles of the flexor and extensor compartment with extension into the tendon sheaths (Figure 3A-3D).

**Figure 4:**
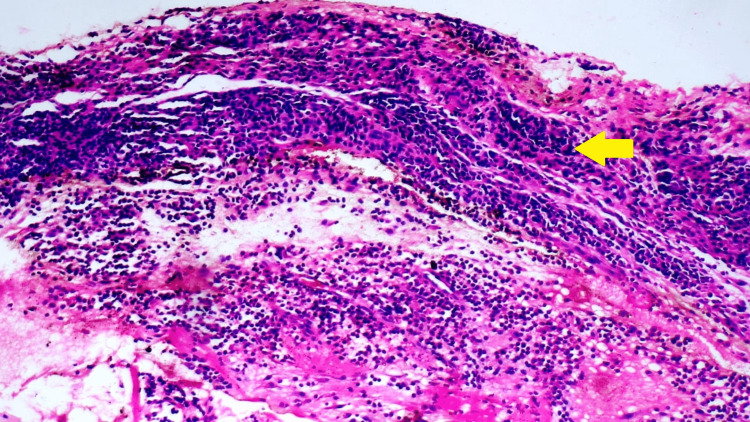
A full-thickness biopsy shows diffuse infiltration of the tissues by lymphocytes and plasma cells (yellow arrow).

The lab investigations revealed an elevated white blood cell count of 13,000 per mm^3^ (normal range, 4000-11000 per mm^3^), an elevated erythrocyte sedimentation rate of 46 mm/hour (normal range, 0-22 mm/hour), an elevated C-reactive protein of 102 mg/L (normal <10mg/L), and a raised eosinophil count of 1.8 x 10^9^/L (normal range, 0-0.5 x 10^9^/L). The immunoglobulin G count was elevated to 2100 mg/dl (normal range, 900-1500 mg/dl). No other investigations were abnormal. To reach a definite diagnosis, a full-thickness forearm biopsy extending from the skin to the muscle was obtained. The biopsy was suggestive of diffuse lymphocytic and plasma cell infiltration in all the layers from the skin to the muscle (Figure 4).

**Figure 5:**
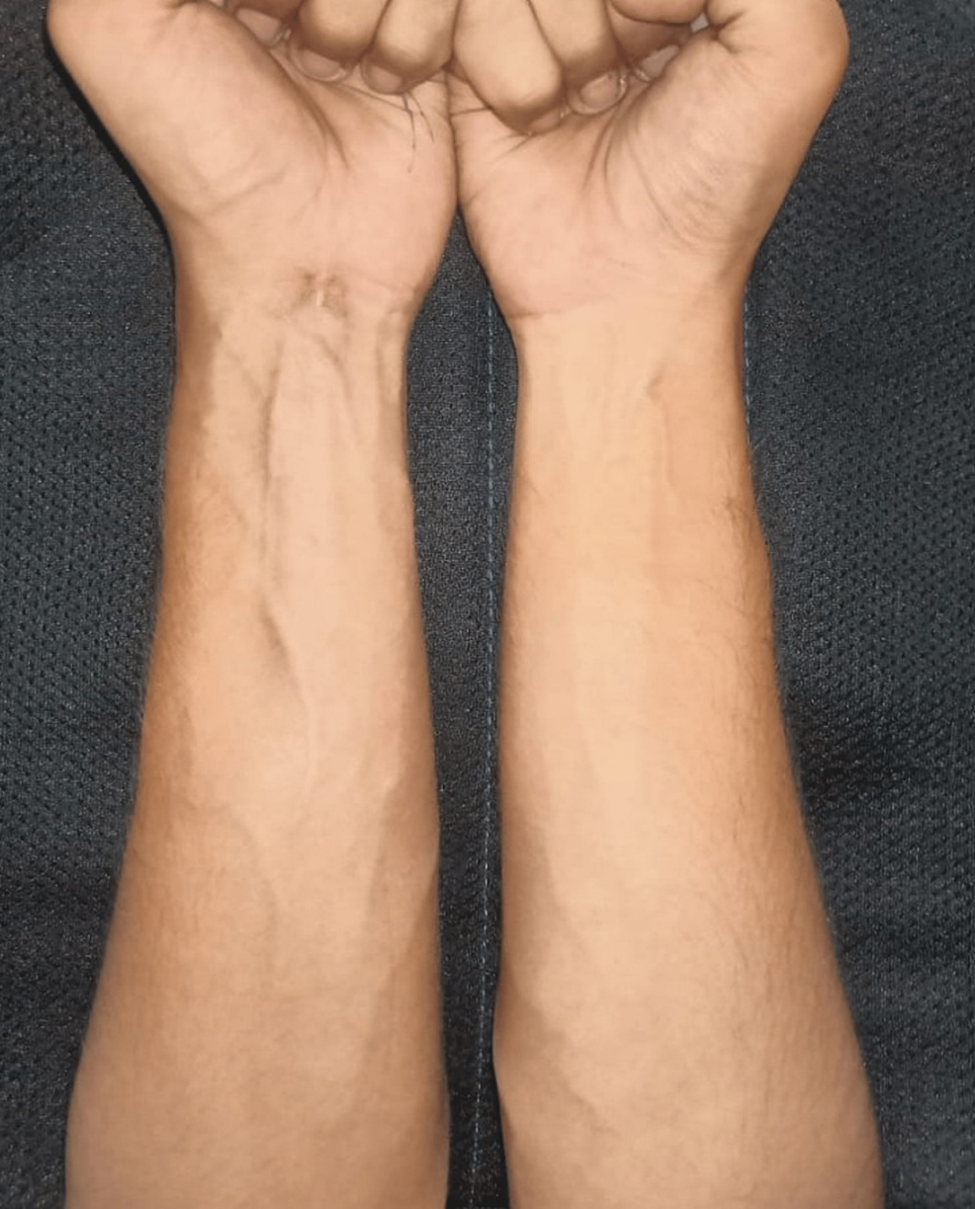
Clinical image taken after initiation of corticosteroid therapy shows a decrease in the swelling and prominence of veins.

A diagnosis of EF was suspected based on the blood parameters and the biopsy picture and the patient was started on oral 40 mg prednisolone for 2 months and was told to follow up every 2 weeks. The patient had improvement in his clinical and laboratory parameters after initiating treatment. The white blood cell count dropped to 10,500/mm^3^, the erythrocyte sedimentation rate fell to 32 mm/hour, and the C-reactive protein level fell to 68 mg/L. The symptoms improved within 5 days as compared to 10 days taken during his first episode after initiation of treatment with non-steroidal anti-inflammatory drugs. In addition to this, the patient also claimed that he had better improvement in the severity of symptoms after starting treatment with prednisolone in comparison to the improvement he had with non-steroidal anti-inflammatory drugs.** **However, he was lost to follow-up due to the COVID-19 pandemic. The patient followed up after around 5 months with a recurrence of the forearm swelling. He stated that he had taken prednisolone for 1 month and then stopped it on his own as he had symptomatic relief. He did not have any flare-ups until his latest episode which occurred 5 months after initiating treatment with prednisolone. He was restarted on oral 40 mg prednisolone and was told to take it strictly for 2 months and follow up every 2 weeks. At a follow-up of 2 months, he was much better clinically and had subsidence of the swelling, erythema, and an improvement in his handgrip (Figure 5).

**Figure 6:**
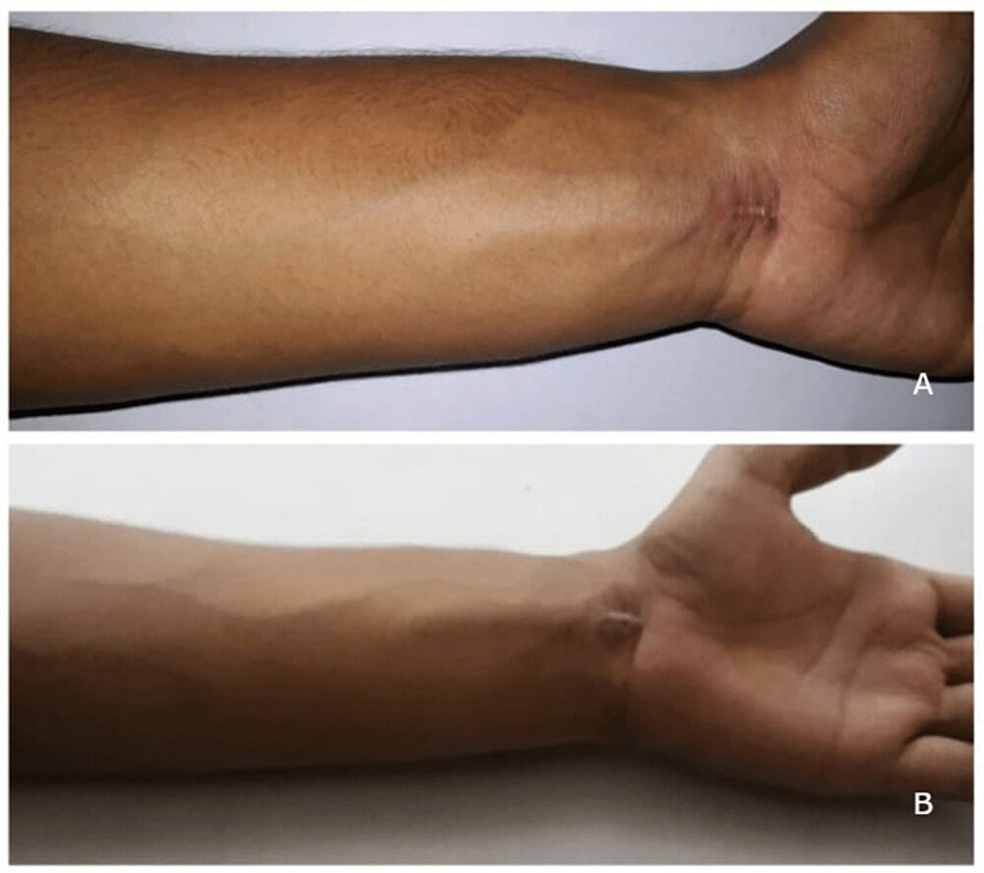
A: Clinical image at the time of aggravation of symptoms. B: Clinical image taken at a follow-up of 4 years shows resolution of the swelling.

His immunoglobulin G count decreased to 1400 mg/dl and the peripheral eosinophil count decreased to 0.8 x 10^9^/L. The dose was tapered to 30 mg for 2 months followed by 20 mg for 2 months. The dose was eventually tapered to 5 mg per day and the patient was told to take it for 5 months and advised to follow up urgently in case of symptom recurrence. The patient did not have any recurrence during this period and treatment was stopped at the end of 5 months. This was accompanied by rigorous physiotherapy to improve the range of motion of the hand and wrist. There was no episode of recurrence after the cessation of treatment. The patient was asymptomatic at the latest follow-up of 4 years and his blood parameters were within normal limits (Figure 6A-6B).

## Discussion

Eosinophilic fasciitis (EF) classically affects adults between 40 to 50 years of age [4]. It starts with symmetrical swelling and erythema of the subcutaneous tissues and skin with the forearms and upper legs involved in a majority of cases [5]. However, our patient had the onset of the disease at a very young age of 18 years with an asymmetrical involvement of the left upper limb. A few typical clinical features associated with EF include muscle weakness, contractures of the joint, carpal tunnel syndrome, and induration of the skin. Pain aggravating when an attempt is made to contract the muscles associated with weakness of the proximal muscles may result in a weak hand grip. Our patient also presented with a weak hand grip on the left side. A well-described clinical finding of EF is the groove sign wherein a linear depression following the vessels in the affected area can be seen on examination.

The etiology of EF is presumed to be multifactorial with possible triggers including strenuous exercise, hemodialysis, certain drugs, and infection with *Borrelia burgdorferi* [6]. Rare associations include recurrent bronchial asthma [7], Hashimoto’s thyroiditis, and aplastic anemia [2]. Rarely, it has also been described as a paraneoplastic syndrome presenting with colorectal carcinoma [8]. However, no risk factor or trigger was evident in our patient. Peripheral eosinophilia is seen in around 60-90% of cases [9], with hypergammaglobulinemia and raised erythrocyte sedimentation rate considered as the other features of the disease. Anti-nuclear antibodies are usually not detected. Our patient also showed similar findings.

A magnetic resonance imaging (MRI) scan can be used for the diagnosis of EF as it can detect the thickening of the fascia and signal abnormalities. A high signal in the fat-suppressed T2W1 sequence is suggestive of EF with a normal low signal representing the normal tissue [10]. MRI proved to be useful in our case as well as it showed evidence of disease. Another investigation used for the detection of thickening of the fascia in patients with EF is local ultrasound. Other than being non-invasive and cheaper than an MRI, it also can be used for monitoring the disease [10]. However, a recent study by Kissin et al. regarding the usefulness of ultrasound in diagnosing EF concluded that the use of ultrasound was limited when compared with magnetic resonance imaging [11]. Hence, we preferred an MRI rather than ultrasound in our case. Moreover, we monitored the patient on the basis of clinical and laboratory parameters instead of ultrasound due to the comparatively increased costs of using ultrasound during each follow-up visit and also because of its operator dependency. Definitive diagnosis rests on the presence of thickened collagen bundles, lymphocytic, and plasma cell infiltration in the full-thickness skin-to-muscle biopsy. The presence of eosinophils is not necessary to make the diagnosis of this disorder with few reports claiming that the degree of eosinophilia doesn’t have any correlation with the severity of the disease process [9]. The biopsy in our patient also revealed a similar picture, with infiltration of lymphocytes and plasma cells.

The various proposed treatment modalities for EF include drugs such as non-steroidal anti-inflammatory drugs, methotrexate, infliximab, and D-penicillamine with corticosteroids considered as the first-line treatment as they are effective in more than 70% of cases [6]. Treatment with corticosteroids is associated with a longer duration of treatment and hence, a few clinicians advocate addition of immunosuppressants at the initial stage of the disease process to reduce the dose as well as the duration of treatment with corticosteroids [10]. Immunosuppressants were not used in our case as the patient showed a prompt and adequate response with the use of corticosteroids alone. Further studies are ongoing currently to determine the efficacy of biological agents in the management of this condition.

Our patient showed a dramatic improvement in his clinical and laboratory parameters after initiating treatment with corticosteroids and did not show any recurrence after treatment cessation. At the latest follow-up of 4 years, he had a significant reduction in the skin induration without the presence of any contractures, a normal range of motion involving the hands and wrist, and normal blood parameters.

## Conclusions

EF is a rare disorder with a greater predilection for the upper extremities and is characterized by inflammation of the skin and subcutaneous tissues. Although various treatments have been proposed for the management of this condition, corticosteroids form the mainstay of management. As there are no clearly described criteria available currently for diagnosing this disease, patients with this condition are usually referred to orthopedics or dermatology. Orthopedic surgeons should thus be aware of this condition to avoid a delay in diagnosis and initiation of treatment.
